# Diet Quality and Health in Older Americans

**DOI:** 10.3390/nu14061198

**Published:** 2022-03-11

**Authors:** Hang Zhao, Tatiana Andreyeva

**Affiliations:** 1 Department of Agricultural and Resource Economics, University of Connecticut, 1376 Storrs Road, Storrs, CT 06269, USA; hang.2.zhao@uconn.edu; 2 Rudd Center for Food Policy and Health, Department of Agricultural and Resource Economics, University of Connecticut, One Constitution Plaza, Hartford, CT 06103, USA

**Keywords:** Healthy Eating Index (HEI), activities of daily living (ADLs), depression, biomarkers

## Abstract

Adequate nutrition is an essential component of healthy ageing. This study documents the quality of diets among older Americans and implications of healthy eating for their physical and mental health. Using a nationally representative longitudinal sample of adults aged ≥50 years, from the Health and Retirement Study (HRS) 2010–2016 and food intake data from the 2013 Health Care and Nutrition Study (HCNS), the study evaluates the onset of health problems along the spectrum of diet quality measured by the Healthy Eating Index (HEI)-2015. Older adults adhering to healthier diets, in the high HEI group, have a significantly lower risk of developing limitations in activities of daily living (15.2% vs. 19.6%, *p* < 0.01) and depression (11.8% vs. 14.9%, *p* < 0.01), as compared to participants with low HEI scores. Consuming healthier diets also predicts more favorable health outcomes, as measured by blood-based biomarkers, including C-reactive protein (3.3 vs. 3.8, *p* < 0.05), cystatin C (1.1 vs. 1.2, *p* < 0.1), total cholesterol (192.1 vs. 196.4, *p* < 0.1), and high-density lipoprotein (57.2 vs. 53.8, *p* < 0.01). Most older Americans can benefit from improving diet to reduce their risk of disability, chronic disease, and depression.

## 1. Introduction

Older Americans comprise of an increasing cohort of the U.S. population, reflecting demographic shifts in the developed world and raising concerns about implications of these changes for society at large [1]. As people get older, they face increasing health challenges, risk the onset of disability, and undergo physiological and social transformations that affect essential elements of their lives, including nutrition. Poor diet is an established predictor of noncommunicable chronic diseases (NCD) and premature mortality, accounting for about 40% of all NCD deaths worldwide [2]. While most Americans need to improve their diets [3], this goal is particularly important for older adults who face additional challenges in maintaining good nutrition. Older adults experience slower metabolism, reduced appetite and energy needs, and impaired mobility, which adversely affect food and nutrient intake [4,5,6,7,8]. In addition to physiological changes, many older adults face a loss of income and earnings that could limit their ability to afford a high-quality diet. Previous research identified financial difficulties as an important risk factor for poor nutrition [9,10]. Social engagement is another factor influencing diet, due to lack of social support, as well as social isolation, which could, for example, restrict access to grocery stores and create difficulties with meal preparation [6,11,12,13]. As a result of social, health, and economic barriers, many older Americans, up to 90% by some estimates [14], have a diet of suboptimal quality.

In this context, there is compelling need to understand food choices and diet quality among older adults and their role as a modifiable predictor of chronic disease and mortality. Prior research has established important links between various measures of diet quality and health of older people, including cardiometabolic mortality, bone growth, type 2 diabetes, cardiovascular diseases (CVD), and NCDs [15,16,17,18,19]. Due to data limitations, many studies in older populations often focused on specific nutrients, rather than the overall diet quality. Just recently, some scholars began to articulate the relationship between nutrition and the risk of chronic disease and functional limitations [20,21,22,23], suggesting that higher overall diet quality is associated with a decreased risk of all-cause, CVD, and cancer mortality and prevention, as well as a delay of functional limitations [24,25,26,27]. Most current studies in this area, however, use self-reported health measures [22,23], with rare exceptions relying on objective evaluations, such as interleukin-6 [21,28,29]. Longitudinal assessments of the relationship between dietary quality and health in older adults are also limited.

The primary objective of this study is to assess the overall dietary quality and its consequences for the onset of physical and mental health problems among Americans over age 50. Health assessment extends beyond the commonly used metrics of self-reported health, functional limitations, and depression to include objective biomarker-based measures of physiological health and well-being. Using a nationally representative longitudinal sample of older adults, we link unique data on food consumption and diet with health sensitive information on five blood-based biomarkers, functional limitations of activities of daily living (ADL), and depression, in order to assess the onset of health problems across the dietary spectrum in people ages over 50. We assess the intake of core food groups and nutrients to construct the Healthy Eating Index (HEI)-2015 that measures the overall diet quality, highlighting the correlates of poor nutrition in older age. We then assess the onset of functional limitations, depression, and poor health, based on biomarkers, to establish the role of diet quality in influencing the risk of developing chronic disease, mental health issues, and physical disability.

## 2. Materials and Methods

### 2.1. Data and Sample Selection

The Health and Retirement Study (HRS) is an ongoing nationally representative longitudinal study of Americans (ages 50 and above) and their spouses, which has been tracking information about important components of older adults’ lives since 1992. Funded by the National Institute on Aging and, with support from the Social Security Administration, the HRS has collected rich longitudinal data to enable research in support of policies on retirement, health care insurance, savings, and economic well-being [30]. Through biannual interviews and additional periodic supplements, the survey collects information about demographics, income and assets, physical and mental health, insurance, family transitions, health care utilization and costs, housing, labor force participation, and employment history. Additional details on the HRS are available elsewhere [30].

Detailed food consumption and nutrient intake was assessed within the HRS panel, through a cross-sectional survey of the 2013 Health Care and Nutrition Study (HCNS), which targeted a sub-sample of the HRS participants. The HCNS included questions about health care access, food purchases, food consumption and nutrition, and food insecurity. In addition to these measures in the core dataset, the HCNS provided a supplementary dataset, with calorie and nutrient totals for 215 nutrient variables, derived from the HCNS Food and Nutrition Section. The HCNS was based on the Harvard University food frequency questionnaire, and estimates of nutrient intake were based on the nutrient tables provided by the Harvard School of Public Health [31,32,33].

Since 2006, the HRS has collected data on blood-based biomarkers every four years for each participant consenting to blood work. The HRS used the dried blood spot (DBS), in which participants agree to have their fingers pricked and have spots of blood dripped onto cards [34]. Then, the blood spots are placed in special foil envelopes with a desiccant packet and then within mailing containers to be shipped to laboratories for analysis [34]. Approximately half of the HRS sample was recruited to complete DBS work in 2006, while the second half of the HRS households provided biomarker data in 2008. The first group, again, gave repeat blood samples in 2010 and 2014, and the second HRS sub-sample did so in 2012 and 2016. These data are considered health sensitive, and they are available to researchers through a data access agreement and request from the University of Michigan [34].

**Figure A1:**
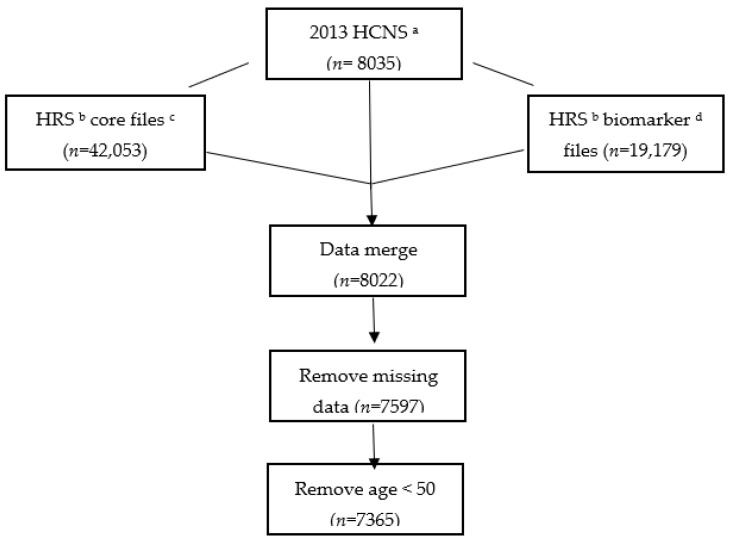
Dataset frame. ^a^: 2013 Health Care and Nutrition Study (HCNS); ^b^: Health and Retirement Study (HRS); ^c^: HRS core files include data from year 2012 and 2014; ^d^: HRS biomarker files include 2010, 2012, 2014, and 2016 HRS biomarker datasets.

Our analytic sample was restricted to the HRS participants in the 2013 HCNS dietary assessment, which we linked to the 2012 and 2014 HRS core files with socio-demographic and self-reported health data, as well as to 2010, 2012, 2014, and 2016 HRS biomarker data. After merging all datasets, we had 8022 participants (see Appendix A Figure A1). As the HRS is a national panel of individuals over age 50, we have excluded participants younger than 50 years of age and those with missing data; we were left with 7365 participants for analyses. Respondents who were excluded were more likely to be old, male, white, and have higher income. The HRS data in our study are publicly available (with a data access agreement for biomarker data as noted above).

### 2.2. Measures

#### 2.2.1. Diet Quality

Diet quality is measured using the Healthy Eating Index (HEI)-2015, which is standard tool to evaluate individual diet, according to its conformance with the key recommendations of the *2015–2020 Dietary Guidelines for Americans* [3]. The HEI-2015 score is based on a 0–100 scoring system and was constructed for this study using the information on food intake in the 2013 HCNS. We distinguished foods into thirteen dietary components and assigned scores to each component, as seen in Table 1, including: total fruit, whole fruit, total vegetables, greens and beans, whole grains, dairy, total protein foods, seafood and plant proteins, fatty acids, refined grains, sodium, added sugars, and saturated fats. Based on the frequency of reported food intake, we assessed each dietary component according to dietary recommendations of the USDA’s ChooseMyPlate [35] and HEI-2015 scoring algorithm [36]. We constructed two groups, including the adequacy and moderation components, with higher scores in the adequacy group reflecting higher diet quality, with higher scores for moderation foods indicating lower diet quality.

We further calculated the density amounts, reflecting the food intake per 1000 kcal, for each diet component. We compared the thirteen diet components with the HEI standard maximum/minimum scores and obtained a total individual HEI score by adding all component scores. For example, based on the USDA standards for whole fruit, if a respondent’s whole fruit consumption was over 0.4 cup per 1000 kcal, he/she would receive a maximum score of 5 for the whole fruit component; if the intake was 0.2 cup, the score would be 2.5. We used the distribution of the HEI-2015 scores to create three tertiles, or groups, of the HRS participants, based on their diet quality: a low (HEI-2015 score < 61), medium (61 ≤ HEI-2015 score ≤ 70), and high HEI group (HEI-2015 score > 70). Higher HEI scores reflect better diet quality.

#### 2.2.2. Health Assessment

Disability assessment was based on a set of questions evaluating limitations of activities of daily living (ADL), including six areas: bathing, dressing, eating, getting in/out of bed, walking across a room, and using the toilet [37]. Participants reporting some difficulty with performing at least one of these six daily tasks were identified as having functional limitations or experiencing ADL-based disability. In addition to the indicator for ADL disability, or having at least one limitation, the study also considered the number of all functional limitations reported to measure the severity of ADL disability. Furthermore, to measure physical health, we have added a count of doctor-diagnosed health conditions (ever told by a doctor of having a respective disease), including high blood pressure, diabetes, cancer, lung disease, heart disease, stroke, psychiatric problems, and arthritis [30].

For depression, the study relied on the Center for Epidemiologic Studies Depression Scale (CES-D) to assess mental health and screen for depression in the HRS participants [38]. Eight depression-related questions were asked in 2012 and 2014, including six negative indicators and two positive indicators. Negative indicators reflected the following symptoms: feeling depressed, feeling everything was an effort, sleep was restless, feeling alone, feeling sad, and could not get “going”. The positive indicators assessed if respondents felt happy and enjoyed life most of the time. If respondents reported four or more negative symptoms, they were classified as experiencing symptoms of depression [39]. 

The study used five blood-based biomarker metrics, available in the HRS, including glycosylated hemoglobin (HbA_1c_), C-reactive protein (CRP), high-density lipoprotein (HDL), total cholesterol, and cystatin C. According to clinical guidelines, HbA1c is an indicator of glycemic control over the past 2–3 months, and excessive HbA1c levels can be a precursor of diabetes. CPR is a general marker of systemic inflammation in the body that is linked to multiple NCDs. Low HDL and high total cholesterol are indicators of lipid levels that can increase the risk of cardiovascular problems. Cystatin C is a measure of kidney functioning [40]. Multiple biomarker measurements can help fully estimate changes in health. 

A series of demographic and socioeconomic characteristics were used as covariates to improve the precision of our estimates. We included the following control variables: gender, age, race/ethnicity, ratio of family income to poverty level, education, body weight status (based on self-reported body weight/height and standard body mass index thresholds), participation in the Supplemental Nutrition Assistance Program (SNAP), marital status, and daily energy intake or calories consumed per day. In addition, baseline measures of the estimated health indicators were included, so that the model measures the relationship between diet quality and the *change* in health status or onset of a health condition, therefore providing a more plausible causal assessment.

### 2.3. Analyses

We used logistic regression models for categorical measures of depression and ADL-based disability, a Poisson regression for the number of ADL functional limitations and count of doctor-diagnosed health conditions, and linear regression models for continuous biomarker indicators. The model was specified as:

$$Y_{it+1}=\beta_{0}+\beta_{1}HEI_{it}+\beta_{2}X_{it-1}+\beta_{3}y_{it-1}+\varepsilon_{it-1}$$

where $Y_{it+1}$ is the health outcome for respondent *i* in year *t* + 1, $HEI_{it}$ is the diet quality group in year *t*, $X_{it-1}$ is a vector of individual level covariates for respondent *i* in year *t* − 1, $y_{it-1}$ is an outcome measure in prior wave, and $\varepsilon_{it-1}$ is the error term. $\beta_{1}$ estimates the relationship between the HEI group and health outcomes. All analyses were weighted to account for the HRS complex survey design, in order to make estimates nationally representative of Americans ages 50 and above.

## 3. Results

Table 1 shows the mean of thirteen dietary components and average HEI-2015 score. The respondents in our sample had an average HEI-2015 score of 65.8, with a median of 66.5 and interquartile range (IQR) of 13.5, suggesting relatively poor diet for most older adults in our analytic sample. Females, Hispanic, normal weight, older adults, and participants of higher socioeconomic status had on average higher HEI-2015 scores (Appendix A Table A1).

### 3.1. Demographic Characteristics

Table 2 includes descriptive statistics of the demographic characteristics and health outcomes, across the HEI groups, representing diet quality. Education was positively correlated with diet quality in older adults: the higher HEI groups had a higher percentage of participants with a college degree (28.4% vs. 17.2%, 24.2% vs. 17.2%, *p* < 0.01) and graduate school and above (14.8% vs. 5.5%, 9.7% vs. 5.5%, *p* < 0.01). Household income had similar trends, with more higher-income households in the high HEI/good diet group (62.5% vs. 44.6%, 53.9% vs. 44.6%, *p* < 0.01) and fewer low-income households in the high HEI group (10.0% vs 19.5%, 12.8% vs 19.5%, *p* < 0.01). Similarly, more respondents receiving SNAP benefits were in the lower-level HEI groups (13.3% vs. 8.1% and 5.7%, *p* < 0.01). Females were more likely than males to have better diet, either in the high or medium HEI groups (59.7% vs. 48.6%, 54.5% vs. 48.6%, *p* < 0.01). Finally, as expected, diet quality was negatively associated with body weight status, with significantly higher rates of obesity in older adults in the low vs high HEI groups (37.4 vs. 31.7%, *p* < 0.01). The breakdown of participants by race/ethnicity was similar across all HEI groups.

### 3.2. ADL Disability, Depression, and Biomarker Indicators across HEI/Diet Quality Groups: Unadjusted Results

As Table 2 reports, HRS participants with higher diet quality had a lower count of doctor-diagnosed health conditions (1.9 vs. 2.3, *p* < 0.01), were significantly less likely to have ADL-based disability (12.5% vs. 22.8%, *p* < 0.01 and 17.0% vs. 22.8%, *p* < 0.01), had lower total number of ADL functional limitations (0.5 vs. 0.4 *p* < 0.01 and 0.5 vs. 0.3, *p* < 0.01), and had lower rates of depression (10.1% vs. 18.4%, *p* < 0.01 and 13.3% vs. 18.4%, *p* < 0.01), as compared with the low HEI group. Better diet quality in older age was also associated with a lower risk of developing type 2 diabetes, hypertension, cardiovascular disease, and poor kidney function, as gauged by blood-based biomarker metrics, including lower levels of glycosylated hemoglobin (HbA_1c_), C-reactive protein, and cystatin C: respectively, 5.8 vs. 5.9, *p* < 0.1; 2.9 vs. 4.2, *p* < 0.01; and 1.07 vs. 1.2, *p* < 0.01. While there was no significant variation in the levels of total cholesterol across the HEI groups, participants with better diet quality had higher levels of CVD-protective, high-density lipoprotein (HDL) than older adults in the poor diet/low HEI group (57.7 vs. 53.5, *p* < 0.01). 

### 3.3. Model Estimation: Predicted Probabilities of ADL Disability, Depression, and Biomarker Health Indicators

Table 3 shows the predicted probabilities of health outcomes across the HEI/ diet quality groups, including the overall sample and gender-specific estimates. Respondents in the high HEI group had the lowest probability of ADL-based disability (15.2% vs. 19.6%, *p* < 0.01) and depression (11.8% vs. 14.9%, *p* < 0.01), after controlling for having these conditions at baseline and socio-demographic covariates. The number of ADL functional limitations, as a proxy for the severity of ADL-based disability, was, on average, lower among participants in the high vs. low HEI groups (0.33 vs. 0.41, *p* < 0.01). 

For the biomarker health metrics, there were significant differences between the highest and lowest HEI groups for C-reactive protein (3.3 vs. 3.8, *p* < 0.05), HDL (57.2 vs. 53.8, *p* < 0.01), total cholesterol (192.1 vs. 196.4, *p* < 0.1), and cystatin C (1.13 vs. 1.17, *p* < 0.1). It should be noted, however, that the level of significance for estimates on total cholesterol and cystatin C were marginal at 10%. No difference by diet quality was observed for glycosylated hemoglobin (HbA_1c_). 

Important gender differences emerged for select health outcomes. For example, while ADL-based disability rates were higher among females vs. males, across all diet groups, particularly large differences in ADL disability were seen for females in the high vs. low HEI group (16.5% vs. 22.5%, *p* < 0.01), with modest differences for males. The health conditions and count of ADL functional limitations varied significantly across the HEI groups for only females (and not males), further supporting stronger diet-related results on health conditions and disability in females. Similarly, depression rates were not linked to diet quality in males, but large differences, across the HEI groups, were seen for females.

In contrast, the benefits of good quality diet appeared to be larger for males for high-density lipoprotein, also known as “good” cholesterol, 54.0 vs. 49.7, *p* < 0.01, vs. a more modest difference in females of 59.8 vs. 57.4, *p* < 0.05. Of note, females in the low HEI group had higher levels of good cholesterol than males with the best diet, suggesting higher CVD risk for males. Total cholesterol did not vary by diet quality for males, despite a significant negative association seen in females (201.4 vs. 195.8, *p* < 0.05). Finally, there were significant differences across the HEI groups for males, but not females, on C-reactive protein (2.8 vs. 3.4, *p* < 0.1).

## 4. Discussion

The primary aim of this study on diet quality among older Americans was to assess correlates of poor nutrition in older age and establish the role of diet quality in the onset of physical disability, depression, and chronic disease. Findings of the study show that diet quality, among most adults ages 50 and above, needs significant improvement, with the average HEI-2015 score of 65.8 reflecting suboptimal diet. People living in households with low income, those with higher BMI, and males have, on average, even lower diet quality. Reassuringly, our estimate of the average HEI-2015 score is almost identical to the estimate for older adults based on the nationally representative data in *What We Eat in America* [41]. This national comparison of diet quality scores for 2013–2014 concluded that the HEI-2015 score was 59 out of 100 for all ages and 64 for older adults, which is a better diet quality than average, yet still well below optimal levels [41].

Certain population groups (e.g., low income, people with low education, divorced, and men) have particularly low diet quality due to important social, cultural, and economic barriers in securing access to good nutrition. More older women than men had higher quality diet in this analysis, which is consistent with previous studies, suggesting that men often face challenges with maintaining good nutrition, potentially due to limited cooking skills, lack of knowledge about good nutrition, or unfavorable attitudes towards healthy diets [6,9]. Men may place greater weight on the pleasure associated with unhealthy diet choices and psychologically be more likely to take risk and/or want to live for the moment, whereas women may be more long-term oriented and worried about health as a long-term concern [42]. 

Prior studies have consistently reported on the negative correlation between diet quality and low educational attainment and income [9,43]. Government policies targeting older adults at particular risk for poor nutrition are an important response to correct this inequity. In the US, there are effective food assistance programs, such as SNAP, that have been shown to reduce food insecurity and contribute to better nutrition, but their uptake among low-income elderly has been less than optimal [44]. Additional effort is needed to promote better nutrition among men and older adults with depression and/or disability, especially those living alone. With increased social isolation, as well as the economic and mental stresses of recent years, many older adults face an even greater risk of poor nutrition and food insecurity. Future policy efforts should focus on identifying effective approaches to improving nutrition among the most vulnerable groups among the elderly. 

In addition to describing the status of diet quality among older adults, this study predicts the risk of developing chronic disease, disability, and depression across the diet spectrum. Our findings show that older people with better diet quality have a significantly lower risk of developing depression and ADL-based disability. As our longitudinal analysis controls for the baseline status of these conditions, we effectually show the beneficial *change* in ADL disability and depression that is associated with better dietary patterns. Such longitudinal assessments are rare and include evidence of the reduced disability [45] and prevention or delay of mobility limitations that is associated with better diet quality [24,26,27].

To our knowledge, this is the first attempt to assess the link between diet quality and biomarker-based health metrics. National datasets containing blood samples linked to rich socio-demographic data for thousands of participants became available to social science researchers only in recent years. This study finds that participants with high quality diet have favorable performance on several biomarkers, indicating a reduced risk for cardiovascular disease, such as “good” cholesterol (HDL), and measures of systemic inflammation, such as C-reactive protein. In fact, out of five examined biomarkers, only one—glycosylated hemoglobin (HbA_1c_)—was not shown to have a significant link to diet quality in older adults. This is consistent with previous research indicating the positive relationship between good diet quality and health [46]. 

Importantly, we find relatively little variation by diet quality for the change in the count of doctor-diagnosed chronic diseases, especially among older men. This could reflect lower sensitivity of health indicators for doctor-diagnosed health conditions, which are, on average, highly prevalent in older adults. As our analysis evaluates the change in the health outcomes across the HEI/diet groups (controlling for baseline status), the count measure of health conditions might perform poorly in capturing health changes in the severity of disease, development of a future problem, or an existence of a problem that was not diagnosed due to limited access to health care. Biomarkers-based measures might serve as more sensitive indicators of changes in physical health.

Our results also show that, for older women, high diet quality has a more significant impact on ADL disability than it does for older men, whereas the opposite is true for “good” cholesterol and C-reactive protein in men. Comparing with older women, men might have been engaged in more physically demanding work, or work with inherent health risks for a longer period of their lives, and that would presumably affect some of their health independent of diet. On this basis, our findings of a more significant impact of diet for women do make sense. Future research, however, should assess the mediating effects between diet quality and mental health, ADLs, and biomarkers, since these might more fully explain the differential impacts by gender that we find in this study. 

### Strengths and Limitations

Whereas previous studies restricted analyses to low-income older people and those living in specific states or areas [7,47], this study is based on a nationally representative longitudinal panel of older Americans, with ample socio-demographic covariates, rich food intake, and sensitive health data on blood-based biomarkers. We utilized multiple measures of health, including a count of health condition, ADLs, depression, and objective biomarker-based measures, in order to improve the overall precision of our prediction. Previous studies on diet and health have used less diverse health measures [37,38,39]. Another significant strength of the study is its innovative approach of linking dietary intake data to objective biomarker-based measures of health and well-being among older Americans. To our knowledge, no prior research has used nationally representative data on diet quality and biomarker-based assessment of health in the same model. Furthermore, the longitudinal nature of the health data helps us get closer to the causal assessment of the diet effects on health and address the threat of reverse causality. We attempt to do so by including the data on ADL disability, depression, and biomarkers, collected prior to data collection on food intake and diet quality. 

Along with its strengths, the study is subject to several important limitations. First, diet quality is assessed based on food frequency questionnaire, rather than 24-h recalls. However, as our average HEI scores are almost identical to the national estimates, based on 24-h recalls, we do not believe the HRS diet data are a major limitation. Availability of diet assessment data only data availability for 2013 does limit longitudinal analyses and precludes assessments of any recent changes in diet quality of older Americans. Further research should evaluate how recent policy, social, and economic changes, including the COVID-19 pandemic, have affected nutrition among older adults. Future policy efforts will need to help these populations avoid further losses in diet quality, in order to reduce the risk of chronic disease, disability, and depression. 

## 5. Conclusions

Most older Americans can benefit from improving their diet. This paper provides evidence that older people with better diet quality have lower risk of developing ADL-based disability and depression. In addition, the findings from biomarker-based aspects of health assessment illustrate that good diet quality is linked to more optimal levels of C-reactive protein, total cholesterol and cystatin C, and high-density lipoprotein and, therefore, reduced risk for developing chronic disease. Expanding access to good nutrition can benefit older people by helping improve mental health, delay or avoid the onset of disability (and its related loss of independence), and reduce the incidence of chronic disease. Future research should focus on evaluating effective policies to improve nutrition among older adults.

## Figures and Tables

**Table 1:** Healthy Eating Index (HEI)–2015 and component scores.

Component (Score Range)	Standard: Maximum Score	Standard: Minimum Score of Zero	All Respondents Mean (95% CI) (*n* = 7365)
Adequacy			
Total fruit (0–5)	≥0.8 cup equiv. per 1000 kcal	No fruit	3.9 (3.8, 4.0)
Whole fruit (0–5)	≥0.4 cup equiv. per 1000 kcal	No whole fruit	4.5 (4.5, 4.6)
Total vegetables (0–5)	≥1.1 cup equiv. per 1000 kcal	No vegetables	3.7 (3.6, 3.7)
Greens and beans (0–5)	≥0.2 cup equiv. per 1000 kcal	No greens and beans	3.8 (3.7, 3.8)
Whole grains (0–10)	≥1.5 oz equiv. per 1000 kcal	No whole grains	4.4 (4.3, 4.5)
Dairy (0–10)	≥1.3 cup equiv. per 1000 kcal	No dairy	5.5 (5.4, 5.6)
Total protein foods (0–5)	≥2.5 oz equiv. per 1000 kcal	No protein foods	3.4 (3.3, 3.4)
Seafood and plant proteins (0–5)	≥0.8 oz equiv. per 1000 kcal	No seafood or plant proteins	2.4 (2.4, 2.5)
Fatty acids (0–10)	(PUFAs + MUFAs)/SFAs ≥2.5	(PUFAs + MUFAs)/SFAs ≤1.2	3.8 (3.7, 3.9)
Moderation			
Refined grains (0–10)	≤1.8 oz equiv. per 1000 kcal	≥4.3 oz equiv. per 1000 kcal	9.0 (8.9, 9.1)
Sodium (0–10)	≤1.1 g per 1000 kcal	≥2.0 g per 1000 kcal	8.3 (8.2, 8.3)
Added sugars (0–10)	≤6.5% of energy	≥26% of energy	7.4 (7.3, 7.5)
Saturated fats (0–10)	≤8% of energy	≥16% of energy	5.8 (5.8, 5.9)
HEI-2015 score	-	-	65.8 (65.4, 66.3)

All analyses were adjusted using the HRS survey weights. CI, confidence interval.

**Table 2:** Participant characteristics by HEI group/diet quality.

Characteristics, Mean	Low HEI Group	Medium HEI Group	High HEI Group
	Mean (95% CI) (*n* = 2173)	Mean (95% CI) (*n* = 2607)	Mean (95% CI) (*n* = 2585)
Outcomes			
Health, disability, and depression			
Count of health conditions	2.3 (2.2, 2.4)	2.2 (2.2, 2.3)	1.9 *** (1.9, 2.0)
Number of ADL functional limitations	0.5 (0.4, 0.6)	0.4 *** (0.3, 0.4)	0.3 *** (0.2, 0.3)
ADL-based disability, %	22.8 (20.1, 25.5)	17.0 *** (15.5, 18.6)	12.5 *** (11.0, 14.0)
Depression, %	18.4 (16.3, 20.5)	13.3 *** (11.7, 14.8)	10.1 *** (8.5, 11.8)
Biomarker indicators			
Glycosylated hemoglobin (HbA_1c_), %	5.9 (5.9, 6.0)	6.0 * (5.9, 6.1)	5.8 * (5.8, 5.9)
C-reactive protein (CRP), mg/L	4.2 (3.8, 4.7)	3.6 ** (3.3, 4.0)	2.9 *** (2.6, 3.2)
High-density lipoprotein (HDL), mg/dL	53.5 (52.1, 54.8)	54.9 (53.6, 56.3)	57.7 *** (56.4, 59.0)
Total cholesterol, mg/dL	195.0 (191.1, 199.0)	192.8 (189.8, 195.8)	194.2 (191.3, 197.2)
Cystatin C, mg/L	1.20 (1.17, 1.24)	1.17 (1.13, 1.20)	1.07 *** (1.04, 1.11)
Demographic and other covariates			
Daily energy (kcal/100)	20.2 (19.5, 20.9)	18.2 *** (17.8, 18.7)	17.8 *** (17.3, 18.3)
Age, years	65.0 (64.3, 65.8)	66.0 ** (65.3, 66.8)	65.8 * (65.1, 66.5)
Socio-demographic covariates, %			
Gender			
Female	48.6 (46.5, 50.7)	54.5 *** (52.5, 56.6)	59.7 *** (57.5, 61.9)
Male	51.4 (49.3, 53.5)	45.5 *** (43.4, 47.5)	40.3 *** (38.1, 42.5)
Race/ethnicity			
Non-Hispanic white	78.4 (75.0, 81.9)	77.4 (73.8, 80.9)	77.0 (73.5, 80.4)
Non-Hispanic black	10.4 (8.2, 12.6)	10.6 (9.0, 12.2)	10.4 (8.7, 12.0)
Hispanic of any race	7.5 (5.2, 9.8)	8.7 (6.0, 11.5)	9.2 (6.4, 11.9)
Other race/ethnicity	3.7 (2.4, 4.9)	3.3 (2.2, 4.5)	3.5 (2.5, 4.5)
Education			
Less than high school education	17.5 (15.6, 19.4)	13.3 *** (11.3, 15.2)	10.0 *** (8.2, 11.7)
High school or GED	59.8 (56.9, 62.7)	52.8 *** (50.1, 55.4)	46.8 *** (44.2, 49.4)
College	17.2 (14.4, 19.9)	24.2 *** (21.5, 26.9)	28.4 *** (25.9, 30.9)
Graduate degree	5.5 (4.2, 6.8)	9.7 *** (8.2, 11.2)	14.8 *** (13.1, 16.6)
Household’s receipt of benefits from Supplemental Nutrition Assistance Program (SNAP)	13.3 (11.2, 15.3)	8.1 *** (6.3, 9.9)	5.7 *** (4.3, 7.1)
Ratio of family income to poverty threshold			
<1.3	19.5 (16.9, 22.2)	12.8 *** (10.2, 15.4)	10.0 *** (8.1, 11.9)
1.3–3.49	35.8 (33.2, 38.5)	33.3 (31.2, 35.5)	27.6 *** (25.3, 29.8)
≥3.5	44.6 (41.6, 47.7)	53.9 *** (50.6, 57.1)	62.5 *** (59.3, 65.6)
Body weight status			
Underweight	1.9 (1.2, 2.6)	0.5 *** (0.2, 0.8)	1.0 * (0.6, 1.5)
Normal weight	27.1 (24.6, 29.6)	25.1 (22.9, 27.3)	30.4 ** (28.6, 32.2)
Overweight	33.7 (31.4, 35.9)	37.9 ** (35.1, 40.7)	36.8 * (34.5, 39.2)
Obese	37.4 (34.9, 39.8)	36.5 (33.8, 39.3)	31.7 *** (29.0, 34.5)
Marital status			
Married/partnered	61.9 (59.2, 64.5)	67.8 *** (65.7, 70.0)	67.8 *** (65.2, 70.3)
Divorced/separated	16.5 (14.7, 18.3)	13.2 *** (11.7, 14.7)	12.0 *** (10.3, 13.6)
Widowed	14.6 (12.7, 16.4)	14.0 (12.2, 15.8)	14.4 (12.6, 16.2)
Never married	7.0 (5.6, 8.4)	5.0 ** (3.8, 6.2)	5.9 (4.7, 7.1)

Notes: All analyses were adjusted using the HRS survey weights. Two-sample *t*-test *p* value: * *p* < 0.1, ** *p* < 0.05, *** *p* < 0.01. Reference group: low HEI group.

**Table 3:** Regression-adjusted predicted results for health outcomes by HEI/diet quality group.

Variables	Low HEI Group Estimated Marginal Means (95% CI)	Medium HEI Group Estimated Marginal Means (95% CI)	High HEI Group Estimated Marginal Means (95% CI)
Health, Disability and Depression Outcomes			
Count of health conditions			
Overall (*n* = 7140)	2.18 (2.14, 2.22)	2.19 (2.15, 2.22)	2.13 (2.09, 2.18)
Female (*n* = 4165)	2.21 (2.16, 2.26)	2.22 (2.17, 2.28)	2.16 * (2.12, 2.21)
Male (*n* = 2975)	2.15 (2.10, 2.21)	2.14 (2.09, 2.19)	2.10 (2.02, 2.17)
Number of functional limitations			
Overall (*n* = 7140)	0.41 (0.37, 0.45)	0.38 (0.34, 0.42)	0.33 *** (0.28, 0.37)
Female (*n* = 4165)	0.47 (0.41, 0.53)	0.45 (0.39, 0.52)	0.35 *** (0.30, 0.41)
Male (*n* = 2975)	0.33 (0.28, 0.39)	0.29 (0.24, 0.35)	0.29 (0.21, 0.38)
ADL-based disability, %			
Overall (*n* = 7140)	19.6 (17.9, 21.3)	16.4 *** (15.1, 17.7)	15.2 *** (14.0, 16.5)
Female (*n* = 4165)	22.5 (19.9, 25.1)	18.2 *** (16.3, 20.1)	16.5 *** (14.7, 18.2)
Male (*n* = 2975)	16.5 (14.2, 18.8)	14.2 (12.2, 16.1)	13.8 * (11.7, 15.9)
Depression, %			
Overall (*n* = 6867)	14.9 (13.4, 16.4)	13.6 (12.0, 15.3)	11.8 *** (10.0, 13.6)
Female (*n* = 4068)	16.9 (14.5, 19.3)	16.2 (13.9, 18.6)	13.1 *** (11.2, 14.9)
Male (*n* = 2799)	12.7 (10.1, 15.2)	10.5 (8.5, 12.6)	10.2 (7.5, 12.9)
Biomarker Indicators			
Glycosylated hemoglobin (HbA_1c_), %			
Overall (*n* = 4405)	5.90 (5.85, 5.94)	5.95 * (5.91, 5.99)	5.89 (5.86, 5.93)
Female (*n* = 2626)	5.88 (5.81, 5.96)	5.93 (5.88, 5.97)	5.87 (5.81, 5.92)
Male (*n* = 1779)	5.91 (5.85, 5.97)	5.99 * (5.91, 6.07)	5.92 (5.86, 5.99)
C-reactive protein, mg/L			
Overall (*n* = 4591)	3.8 (3.4, 4.3)	3.6 (3.2, 4.0)	3.3 ** (2.9, 3.6)
Female (*n* = 2741)	4.2 (3.5, 4.9)	3.9 (3.3, 4.5)	3.6 (3.3, 3.9)
Male (*n* = 1850)	3.4 (2.9, 3.9)	3.2 (2.7, 3.7)	2.8 * (2.4, 3.3)
High-density lipoprotein, mg/dL			
Overall (*n* = 4369)	53.8 (52.9, 54.8)	55.5 ** (54.3, 56.8)	57.2 *** (56.0, 58.3)
Female (*n* = 2633)	57.4 (56.1, 58.7)	58.2 (56.9, 59.6)	59.8 ** (58.3, 61.2)
Male (*n* = 1736)	49.7 (48.1, 51.4)	52.1 ** (50.4, 53.9)	54.0 *** (52.3, 55.7)
Total Cholesterol, mg/dL			
Overall (*n* = 4586)	196.4 (192.6, 200.2)	193.4 (190.8, 196.0)	192.1 * (189.2, 194.9)
Female (*n* = 2742)	201.4 (197.3, 205.4)	195.7 ** (192.0, 199.5)	195.8 ** (192.8, 198.7)
Male (*n* = 1844)	190.6 (185.7, 195.5)	190.5 (186.5, 194.5)	187.7 (183.1, 192.3)
Cystatin C, mg/L			
Overall (*n* = 4531)	1.17 (1.14, 1.20)	1.13 ** (1.11, 1.15)	1.13 * (1.09, 1.16)
Female (*n* = 2715)	1.16 (1.12, 1.20)	1.14 (1.12, 1.17)	1.13 (1.09, 1.17)
Male (*n* = 1816)	1.18 (1.13, 1.22)	1.12 ** (1.08, 1.15)	1.12 (1.07, 1.18)

Notes: All analyses were adjusted using the HRS survey weights. Reference group: low HEI group. According to the data distribution, logistic regression for categorical variables (depression and ADL disability), Poisson regression for the number of functional limitations of ADL disability and count of doctor-diagnosed health conditions, and a linear regression for continuous variables (biomarkers). Covariates included total daily energy consumed, gender, age, race/ethnicity, education, income ratio, body weight status (based on self-reported height and weight), SNAP benefit receipt, marital status, and health outcomes at baseline. * *p* < 0.1, ** *p* < 0.05, *** *p* < 0.01.

## Data Availability

Requests for access to the HRS data should be submitted through the official website of the Health and Retirement Study.

## Appendix A

**Table A1:** Healthy Eating Index–2015 (HEI-2015) and component scores.

Component (Range)	Female		Hispanic	
	Yes (*n* = 4322)	No (*n* = 3043)	Yes (*n* = 846)	No (*n* = 6519)
Adequacy				
Total fruit (0–5)	4.1 (4.0, 4.1)	3.7 *** (3.6, 3.8)	4.1 (4.0, 4.2)	3.9 *** (3.8, 3.9)
Whole fruit (0–5)	4.6 (4.6, 4.7)	4.4 *** (4.4, 4.5)	4.7 (4.6, 4.8)	4.5 *** (4.5, 4.6)
Total vegetables (0–5)	3.9 (3.8, 4.0)	3.4 *** (3.4, 3.5)	3.9 (3.8, 4.0)	3.7 *** (3.6, 3.7)
Greens and beans (0–5)	4.0 (3.9, 4.1)	3.5 *** (3.5, 3.6)	3.7 (3.5, 3.8)	3.8 (3.7, 3.8)
Whole grains (0–10)	4.4 (4.3, 4.5)	4.4 (4.2, 4.6)	5.7 (5.3, 6.1)	4.3 *** (4.2, 4.4)
Dairy (0–10)	5.7 (5.6, 5.8)	5.2 *** (5.1, 5.3)	5.3 (5.1, 5.5)	5.5 (5.4, 5.6)
Total protein foods (0–5)	3.3 (3.2, 3.3)	3.5 *** (3.4, 3.5)	3.2 (3.0, 3.3)	3.4 *** (3.3, 3.4)
Seafood and plant proteins (0–5)	2.4 (2.4, 2.5)	2.4 (2.3, 2.4)	2.1 (1.9, 2.3)	2.4 *** (2.4, 2.5)
Fatty acids (0–10)	3.7 (3.6, 3.8)	3.8 (3.7, 3.9)	4.1 (3.8, 4.3)	3.7 ** (3.6, 3.8)
Moderation				
Refined grains (0–10)	9.1 (9.0, 9.2)	9.0 (8.8, 9.1)	7.3 (6.8, 7.8)	9.2 *** (9.1, 9.2)
Sodium (0–10)	8.3 (8.2, 8.4)	8.2 * (8.1, 8.3)	8.2 (8.0, 8.5)	8.3 (8.2, 8.3)
Added sugars (0–10)	7.4 (7.3, 7.6)	7.3 * (7.1, 7.4)	7.9 (7.7, 8.1)	7.3 *** (7.2, 7.4)
Saturated fats (0–10)	5.8 (5.7, 5.9)	5.9 (5.8, 6.0)	6.7 (6.5, 6.9)	5.8 *** (5.7, 5.8)
HEI–2015 score	66.7 (66.3, 67.2)	64.7 *** (64.1, 65.3)	66.8 (65.9, 67.7)	65.7 ** (65.3, 66.2)
	**Body Mass Index (BMI)**			
**Component (Range)**	**Normal Weight (*n* = 1920)**	**Obese (*n* = 2566)**	**Overweight (*n* = 2641)**	**Underweight (*n* = 76)**
Adequacy				
Total fruit (0–5)	3.9 (3.8, 4.0)	3.9 (3.8, 3.9)	3.9 (3.8, 4.0)	3.8 (3.2, 4.3)
Whole fruit (0–5)	4.5 (4.5, 4.6)	4.6 (4.5, 4.6)	4.6 (4.5, 4.6)	4.3 (3.9, 4.7)
Total vegetables (0–5)	3.7 (3.6, 3.8)	3.7 (3.6, 3.8)	3.7 (3.6, 3.7)	3.2 ** (2.8, 3.6)
Greens and beans (0–5)	3.8 (3.8, 3.9)	3.8 (3.7, 3.9)	3.8 * (3.7, 3.8)	3.4 ** (3.0, 3.8)
Whole grains (0–10)	4.4 (4.2, 4.6)	4.4 (4.3, 4.6)	4.4 (4.3, 4.6)	3.9 (3.2, 4.6)
Dairy (0–10)	5.5 (5.3, 5.7)	5.4 (5.3, 5.6)	5.5 (5.4, 5.6)	4.9 * (4.2, 5.5)
Total protein foods (0–5)	3.1 (3.0, 3.2)	3.6 *** (3.5, 3.7)	3.3 *** (3.3, 3.4)	2.9 (2.6, 3.3)
Seafood and plant proteins (0–5)	2.4 (2.3, 2.5)	2.4 (2.4, 2.5)	2.4 (2.4, 2.5)	2.2 (1.9, 2.6)
Fatty acids (0–10)	4.0 (3.9, 4.2)	3.6 *** (3.4, 3.7)	3.8 ** (3.6, 3.9)	3.6 (2.7, 4.5)
Moderation				
Refined grains (0–10)	9.1 (9.0, 9.2)	8.9 * (8.8, 9.1)	9.0 (8.9, 9.1)	8.9 (8.4, 9.5)
Sodium (0–10)	8.5 (8.4, 8.7)	7.9 *** (7.8, 8.1)	8.3 *** (8.2, 8.4)	8.9 ** (8.6, 9.3)
Added sugars (0–10)	7.4 (7.2, 7.5)	7.4 (7.3, 7.6)	7.3 (7.2, 7.5)	6.4 *** (5.6, 7.1)
Saturated fats (0–10)	6.0 (5.9, 6.1)	5.5 *** (5.4, 5.7)	6.0 (5.8, 6.1)	6.2 (5.2, 7.2)
HEI-2015 score	66.5 (65.9, 67.1)	65.1 *** (64.5, 65.7)	66.0 (65.4, 66.6)	62.7 ** (59.2, 66.1)
	**Age**			
**Component (range)**	**Age 50–60 (*n* = 2374)**	**Age 61–70 (*n* = 2094)**	**Age 71–80 (*n* = 2026)**	**Age above 81 (*n* = 871)**
Adequacy				
Total fruit (0–5)	3.7 (3.6, 3.8)	3.8 (3.8, 3.9)	4.2 (4.1, 4.3)	4.3 (4.3, 4.4)
Whole fruit (0–5)	4.4 (4.3, 4.5)	4.5 *** (4.5, 4.6)	4.7 *** (4.7, 4.8)	4.8 *** (4.7, 4.9)
Total vegetables (0–5)	3.6 (3.5, 3.7)	3.7 * (3.7, 3.8)	3.7 * (3.7, 3.8)	3.7 (3.6, 3.8)
Greens and beans (0–5)	3.8 (3.7, 3.9)	3.8 (3.7, 3.9)	3.8 (3.7, 3.8)	3.7 (3.6, 3.8)
Whole grains (0–10)	4.4 (4.2, 4.6)	4.4 (4.2, 4.6)	4.5 (4.3, 4.6)	4.3 (4.1, 4.5)
Dairy (0–10)	5.4 (5.3, 5.6)	5.3 (5.1, 5.5)	5.6 ** (5.5, 5.8)	5.9 *** (5.7, 6.2)
Total protein foods (0–5)	3.5 (3.4, 3.5)	3.4 (3.4, 3.5)	3.2 (3.1, 3.3)	3.1 (3.0, 3.2)
Seafood and plant proteins (0–5)	2.4 (2.3, 2.5)	2.5 (2.4, 2.6)	2.4 (2.3, 2.5)	2.3 * (2.1, 2.4)
Fatty acids (0–10)	3.7 (3.6, 3.9)	3.9 (3.8, 4.1)	3.8 (3.7, 4.0)	3.3 (3.1, 3.5)
Moderation				
Refined grains (0–10)	9.1 (8.9, 9.2)	9.0 (8.8, 9.1)	9.1 (8.9, 9.2)	9.0 (8.8, 9.2)
Sodium (0–10)	8.3 (8.1, 8.4)	8.1 * (8.0, 8.2)	8.3 (8.2, 8.4)	8.5 ** (8.4, 8.6)
Added sugars (0–10)	7.2 (7.0, 7.4)	7.6 *** (7.4, 7.8)	7.4 ** (7.3, 7.6)	7.2 (7.0, 7.4)
Saturated fats (0–10)	5.8 (5.6, 5.9)	5.9 (5.7, 6.0)	6.0 ** (5.8, 6.1)	5.8 (5.6, 6.1)
HEI-2015 score	65.2 (64.4, 65.9)	66.1 ** (65.5, 66.6)	66.7 *** (66.0, 67.4)	65.9 (65.1, 66.6)
**Component (range)**	**Ratio of Household Income to the Poverty**			
	**<1.30 (*n* = 1126)**	**1.30–3.49 (*n* = 2692)**	**≥3.50 (*n* = 3547)**	
Adequacy				
Total fruit (0–5)	3.7 (3.6, 3.9)	3.9 ** (3.8, 4.0)	3.9 ** (3.9, 4.0)	
Whole fruit (0–5)	4.4 (4.2, 4.5)	4.6 ** (4.5, 4.6)	4.6 *** (4.5, 4.6)	
Total vegetables (0–5)	3.3 (3.2, 3.5)	3.6 *** (3.5, 3.7)	3.8 *** (3.8, 3.9)	
Greens and beans (0–5)	3.4 (3.3, 3.6)	3.6 ** (3.6, 3.7)	4.0 *** (3.9, 4.0)	
Whole grains (0–10)	4.4 (4.0, 4.8)	4.3 (4.2, 4.5)	4.5 (4.4, 4.6)	
Dairy (0–10)	5.2 (5.0, 5.5)	5.4 (5.3, 5.6)	5.5 ** (5.4, 5.6)	
Total protein foods (0–5)	3.3 (3.2, 3.4)	3.3 (3.3, 3.4)	3.4 * (3.4, 3.5)	
Seafood and plant proteins (0–5)	2.2 (2.1, 2.3)	2.2 (2.2, 2.3)	2.6 *** (2.5, 2.6)	
Fatty acids (0–10)	3.6 (3.4, 3.7)	3.6 (3.5, 3.8)	3.9 *** (3.7, 4.0)	
Moderation				
Refined grains (0–10)	8.3 (7.9, 8.7)	8.9 *** (8.8, 9.1)	9.2 *** (9.2, 9.3)	
Sodium (0–10)	8.4 (8.2, 8.6)	8.2 * (8.1, 8.3)	8.2 (8.1, 8.4)	
Added sugars (0–10)	6.7 (6.4, 7.0)	7.1 ** (6.9, 7.2)	7.7 *** (7.6, 7.8)	
Saturated fats (0–10)	5.9 (5.7, 6.2)	5.8 (5.6, 5.9)	5.9 (5.7, 6.0)	
HEI-2015 score	62.9 (62.1, 63.8)	64.7 *** (64.2, 65.2)	67.2 *** (66.7, 67.8)	

Notes: All analyses were adjusted using the HRS survey weights. CI, confidence interval two-sample *t*-test *p* value: * *p* < 0.1 ** *p* < 0.05 *** *p* < 0.01. Reference group: normal weight, age 50–60, low ratio of household income to the poverty.
